# Mesenchymal Stromal Cells for Sphincter Regeneration: Role of Laminin Isoforms upon Myogenic Differentiation

**DOI:** 10.1371/journal.pone.0137419

**Published:** 2015-09-25

**Authors:** Tanja Seeger, Melanie Hart, Manuel Patarroyo, Bernd Rolauffs, Wilhelm K. Aicher, Gerd Klein

**Affiliations:** 1 University Medical Clinic Department II, Center for Medical Research, University of Tübingen, Tübingen, Germany; 2 Department of Urology, University of Tübingen, Tübingen, Germany; 3 Department of Dental Medicine, Karolinska Institute, Stockholm, Sweden; 4 BG Trauma Clinic, University of Tübingen, Tübingen, Germany; Instituto Butantan, BRAZIL

## Abstract

Multipotent mesenchymal stromal cells (MSCs) are well known for their tri-lineage potential and ability to differentiate *in vitro* into osteogenic, chondrogenic or adipogenic lineages. By selecting appropriate conditions MSCs can also be differentiated *in vitro* into the myogenic lineage and are therefore a promising option for cell-based regeneration of muscle tissue such as an aged or damaged sphincter muscle. For the differentiation into the myogenic lineage there is still a need to evaluate the effects of extracellular matrix proteins such as laminins (LM) which are crucial for different stem cell types and for normal muscle function. The laminin family consists of 16 functionally different isoforms with LM-211 being the most abundant isoform of adult muscle tissues. In the sphincter tissue a strong expression of the isoforms LM-211/221, LM-411/421 and LM-511/521 can be detected in the different cell layers. Bone marrow-derived MSCs in culture, however, mainly express the isoforms LM-411 and LM-511, but not LM-211. Even after myogenic differentiation, LM-211 can hardly be detected. All laminin isoforms tested (LM-211, LM-411, LM-511 and LM-521) showed a significant inhibition of the proliferation of undifferentiated MSCs but, with the exception of LM-521, they had no influence on the proliferation of MSCs cultivated in myogenic medium. The strongest cellular adhesion of MSCs was to LM-511 and LM-521, whereas LM-211 was only a weakly-adhesive substrate for MSCs. Myogenic differentiation of MSCs even reduced the interaction with LM-211, but it did not affect the interaction with LM-511 and LM-521. Since during normal myogenesis the latter two isoforms are the major laminins surrounding developing myogenic progenitors, α5 chain-containing laminins are recommended for further improvements of myogenic differentiation protocols of MSCs into smooth muscle cells.

## Highlights

- Mesenchymal stromal cells (MSCs) mainly express and secrete the laminin isoforms LM-411 and LM-511
- Even after myogenic differentiation LM-211 is hardly expressed by MSCs
- Different laminins inhibit the proliferation of MSCs and do not enhance their myogenic differentiation
- Myogenic differentiation of MSCs reduces their interaction with laminins

## Introduction

Replacement of damaged or aged muscle tissue with adult human stem cells is a highly promising field in regenerative medicine for reviving tissue function [1, 2]. Human mesenchymal stromal cells (MSCs) are multipotent adult stem cells with the capacity to differentiate *in vitro* into the osteogenic, chondrogenic and adipogenic lineages [3, 4]. Under appropriate conditions, MSCs can also differentiate into the myogenic lineage, another cell type of mesodermal origin [5–10]. While MSCs can be found in various adult and embryonic tissues, most studies and clinical applications were performed with bone marrow- or adipose tissue-derived MSCs [11]. However, since the differentiation capacity of the heterogeneous MSCs populations of different origin can substantially vary [12, 13], optimal conditions for the desired differentiation pathway have to be established for each cell population.

Stress urinary incontinence is a pathological condition characterized by the decline of muscle cells in the urethral sphincter apparatus and replacement of the myogenic cells by connective tissue cells [14]. The urinary sphincter consists of different muscle cell layers including smooth muscle cells and a striated muscle cell layer. The inner part, the so-called lissosphincter, surrounds the urethra and is formed by circular and longitudinally oriented smooth muscle cell layers. The lissosphincter is encircled by a horseshoe-shaped striated muscle cell layer known as the rhabdosphincter [15]. A functional integrity of both tissues, the lissosphincter and the rhabdosphincter, is needed for urinary continence. A cell-based therapy using myogenically differentiating cells shows great promise for regenerating or rebuilding a functional sphincter muscle [11, 16]. Since MSCs are able to differentiate into various lineages of mesodermal origin, it is deemed advantageous to pre-differentiate the MSCs *in vitro* into the myogenic lineage, thus avoiding unwanted differentiation processes in the damaged tissue [3].

Smooth muscle cells, alongside skeletal muscle cells, are myogenic cells surrounded by a basement membrane, a highly structured extracellular matrix sheet containing a laminin network as an essential building block [17]. Laminins are a family of heterotrimeric molecules each consisting of an α,β and γ chain. Eleven human laminin chains (five α, three β and three γ chains) have been identified and characterized giving rise to at least sixteen unique isoforms with different biological activities [18, 19]. Laminins, especially the isoforms LM-511 and LM-521, play an important role in the self-renewal of embryonic stem cells or induced pluripotent stem cells [20]. These two laminin isoforms seem to be the natural laminin isoforms for most adult stem cells [19]. During the myogenic process LM-511 and LM-521 are the major laminin isoforms surrounding newly formed muscle cells [21]. The laminin isoform LM-211, which alongside LM-511 is the major isoform of adult skeletal muscle tissues, has also been shown to be functionally involved in smooth muscle cell generation [22, 23]. For these reasons it seemed likely that laminin isoforms could facilitate the differentiation of bone marrow-derived MSCs into myogenic cells. Therefore the aim of our study was to analyze the influence of distinct laminin isoforms on the myogenic differentiation process of human bone marrow-derived MSCs.

## Materials and Methods

### Human primary cells and cell lines

For the isolation of human MSCs, bone marrow taken from the proximal femur during routine hip replacement was obtained from the BG Trauma Clinic (Tübingen) after written consent of patients and approval of the ethics committee of the Medical Faculty of the University of Tübingen (reference number 453/2011/BO). Bone marrow cells were washed once with Dulbecco's phosphate-buffered saline (DPBS; Gibco Life Technologies, Darmstadt, Germany) to remove the fat layer and diluted 1:2 in DPBS. Mononuclear cells were isolated by Histopaque (1.077 g/ml; Sigma-Aldrich, Taufkirchen, Germany) density gradient centrifugation and washed with DPBS. The isolated cells were seeded into T75 cell culture flasks and the medium was exchanged after 24 h to remove non-attached cells. The adherent MSCs were cultivated in expansion medium (‘GMP+ medium’) consisting of DMEM low glucose (Lonza, Basel, Switzerland) with 5% fresh frozen plasma (TCS Bioscience, Buckingham, United Kingdom), 5% human thrombocyte lysate (Blood Donation Center, University of Tübingen), 2 mM L-glutamine (Lonza, Basel, Switzerland), 1000 IE heparin sodium salt (Roth, Karlsruhe, Germany) and 25 mM HEPES sodium salt solution (Sigma-Aldrich).

Primary human bladder-derived smooth muscle cells (HBdSMC; PromoCell, Heidelberg, Germany) cultured in smooth muscle growth medium (PromoCell) and the human smooth muscle cell line HITB5 (Cellutions Biosystems, Burlington, Ontario, Canada) cultured in SmGM^TM^ smooth muscle growth medium-2 plus SingleQuots^TM^ Kits (Lonza) were used as controls for human smooth muscle cell types.

### Myogenic differentiation of mesenchymal stromal cells

MSCs were cultivated in DMEM high glucose (Invitrogen Life Technologies, Darmstadt, Germany) with 10% fetal bovine serum (FBS; Biochrom, Berlin, Germany), 5 ng/ml transforming growth factor-β1 (TGF-β1; R&D Systems, Wiesbaden, Germany), 5 ng/ml platelet derived growth factor-AB (PDGF-AB; Peprotech, Hamburg, Germany) and 30 μM ascorbic acid (Sigma-Aldrich) for at least seven days to induce smooth muscle cell differentiation as recently reported [24]. The myogenic differentiation medium (‘Myo’) without the growth factors and ascorbic acid was used as a control medium (‘CM’).

### Recombinant proteins and antibodies

The human recombinant laminin isoforms LM-211, LM-411, LM-421, LM511 and LM-521 were obtained from Biolamina (Stockholm, Sweden). The following monoclonal and polyclonal antibodies were used to detect the different laminin chains: clones 6C3 and 3H2 against the human α4 chain [25], clones 4B5 and 4B12 against the human α5 chain [25, 26], rat anti-mouse laminin α2 chain (clone 4H8-2, kindly provided by Prof. Lydia Sorokin, University of Münster, Germany), rat anti-mouse laminin β1 chain (Abcam, Cambridge, UK), and clones C4 and D18 against the laminin β2 and γ1 chains, respectively (both from the Developmental Studies Hybridoma Bank, Iowa, USA). The antibodies against the myogenic marker molecules calponin and α-smooth muscle actin (αSMA) were obtained from Abcam, the antibody against transgelin from Santa Cruz Biotechnology (Heidelberg, Germany) and the antibody against human vinculin from Sigma-Aldrich. For staining of endothelial cells, the antiserum against von Willebrand factor (vWF) was obtained from DAKO (Eching, Germany). The monoclonal antibodies against the different integrin chains were as follows: clone P1B5 against the integrin α3 chain (Merck Millipore, Darmstadt, Germany), clone GoH3 against the integrin α6 chain (BD Pharmingen, Heidelberg, Germany), mouse anti-human integrin α7 chain (Abcam) and clone 4B4 against the integrin β1 chain (Beckman Coulter, Krefeld, Germany).

### Reverse transcriptase-polymerase chain reaction analysis

Total RNA was isolated from cell pellets using Qiashredder and Qiagen RNeasy Mini Kit (Qiagen, Hilden, Germany). The cDNA synthesis (SuperScript^TM^ III First-Strand Synthesis System; Invitrogen Life Technologies) was performed using 1 μg total RNA. For amplification of the target cDNA, REDTaq® ReadyMix^TM^ PCR Reaction Mix (Sigma-Aldrich) was used following the instructions of the manufacturer. Primer pairs were selected according to the sequences published in the GenBank database. As a positive control, a primer pair for β-actin was used. First, cDNA was denatured for 45 sec at 95°C, then temperature cycling (29 cycles) was performed: denaturation at 95°C for 45 sec, annealing at 58°C (laminin chains) / 60°C (integrin chains) for 40 sec and elongation at 72°C for 60 sec. Final elongation at 72°C for 10 min terminated the temperature cycling. Samples were then loaded onto a 2% agarose gel (SeaKem® LE Agarose; Lonza) and stained with GelRed^TM^ (Biotium, Hayward, CA, USA). Amplified products were analyzed by exposure under ultraviolet light.

For the quantitative determination of αSMA, calponin and transgelin mRNA expression (QuantiTect Primer Assay; Qiagen), quantitative RT-PCR (qRT-PCR) was performed by using SYBR Green (SYBR Green I LightCycler® 480 Master; LightCycler® 480 Instrument; Roche, Mannheim, Germany) using the following protocol: denaturation at 95°C for 5 min followed by repeated temperature cycling (39 cycles) of denaturation at 95°C for 10 sec, annealing at 62°C for 20 sec and elongation at 72°C for 30 sec. The samples were normalized to the housekeeping genes glyceraldehyde 3-phosphate dehydrogenase (GAPDH; Biomol, Hamburg, Germany) and peptidylprolyl isomerase A (PPIA; Biomol) according to the MIQE guidelines [27]. The melting curve was performed after preheating the samples to 95°C for 0.5 sec. To determine relative expression levels of the genes of interest (GOI) cycle thresholds (C_T_) of the individual genes were compared with those of the housekeeping genes (HG) to determine the relative expression levels. Relative fold changes between the expression of the GOI in undifferentiated and myogenically differentiated MSCs samples were determined by the following equation: fold change = E_GOI_^[ΔC^_T_^GOI]^ / E_HG_^[ΔC^_T_^HG]^, where E = PCR reaction efficacy and [ΔC_T_ GOI] = (C_T untreated_−C_T treated_)_GOI_; [ΔC_T_ HG] = (C_T untreated_−C_T treated_)_HG_.

### Hematoxylin-eosin staining

Sphincter tissue was taken from healthy mini-pigs. Prior to sacrifice animals were sedated (Azaperon, Atropin), then given anaestesia by Propofol (4 mg/kg) and Isofluran (1 Vol%), followed by euthanasia through a lethal dosis of KCl i.v. provided by a veterinarian. Death was confirmed by the veterinarian prior to harvesting samples. The use of animal samples was approved by the Regierungspräsidium Tübingen (file # CU1/12). The dissected tissue was frozen in Tissue-Tek OCT compound (Sakura, Staufen, Germany) and stored at –70°C until used. 5 μm cryostat sections were fixed with acetone for 10 min at -20°C and air-dried. The sections were soaked with ddH_2_O for 5 min and stained with Mayer's hemalaun solution (Merck Millipore) for 5 min, rinsed under tap water for 5 min and then stained with aqueous 0.1% eosin G solution (Merck Millipore). The sections were rinsed under ddH_2_O and dehydrated with an ascending alcohol series (70%, 96%, 100% ethanol, xylene) each for 5 min and mounted with entellan (Merck Millipore).

### Immunofluorescence staining

Undifferentiated MSCs and the myogenic cell types were seeded in eight-well chamber slides (Sarstedt, Nümbrecht, Germany) and cultivated to 90% confluence. The adherent cells and the cryostat sections of sphincter tissue were fixed with methanol for 5 min at -20°C and washed with DPBS. The cells and tissue samples were incubated for 1 h with the primary antibodies diluted in DPBS containing 0.1% bovine serum albumin. After washing with DPBS, bound antibodies could be detected by Cy2-/ Cy3-conjugated goat anti-mouse, anti-rabbit or anti-rat antibodies, respectively. By counterstaining with 0.5 μg/ml 4’,6-diamino-2-phenylindol-dihydrochloride (DAPI) cell nuclei were identified. For control staining the first antibodies were omitted. Slides were mounted with DAKO fluorescence mounting medium (DAKO) and photographs were taken on a Zeiss Axiophot microscope.

### Western blot analysis

Mini-pig bladder tissue was obtained from pigs immediately after being sacrificed. Tissue and cells were lysed using RIPA lysing buffer, consisting of 40 mM Tris base, 150 mM sodium chloride, 1% Nonidet P-40, 0.5% sodium deoxycholate, 0.1% sodium dodecyl sulfate and 2 mM EDTA, for 1 h at 4°C. Immunoprecipitated cell culture supernatants, recombinant proteins, cell lysates and tissue lysates were diluted in sample buffer containing 0.2 M dithiotreitol and 12% β-mercaptoethanol and boiled for 5 min at 95°C. Samples were separated on NuPAGE 3–8% Tris-acetate gels (laminin chains; Life Technologies) / 10% polyacrylamide gels (calponin and αSMA) / 10% Bis-Tris (transgelin; Life Technologies), respectively, using Tris-acetate running buffer (laminin chains) consisting of 50 mM tricine, 50 mM Tris base and 0.1% sodium dodecyl sulfate or running buffer (calponin, αSMA, transgelin) consisting of 25 mM Tris base, 96 mM glycine and 3.5 mM sodium dodecyl sulfate. Polyvinylidene difluoride membranes (PVDF; Merck Millipore) were submerged in methanol and proteins were transferred by wet blotting (laminin chains) using transfer buffer containing 25 mM Tris base, 192 mM glycine and 5% methanol or semidry blotting (calponin, αSMA, transgelin) using 10 mM N-cyclohexyl-3-aminopropanesulfonic acid buffer (CAPS). All membranes were blocked in Tris-buffered saline containing 0.1% Tween-20 and 5% skimmed milk powder (Roth), incubated with the primary antibodies overnight at 4°C and the secondary antibodies for 1 h at 37°C. Bound antibodies were detected using the WesternSure Chemiluminescent Substrates (LI-COR, Lincoln, Nebraska, USA) or NBT/BCIP tablets (Roche). Equal loading could be ensured by stripping the blots and reprobing with the anti-vinculin antibody.

### Flow cytometry

Expression of the integrin α7 chain (ITGA7) was studied by flow cytometry. All incubation steps were performed at 4°C. 2x10^5^ cells were incubated for 10 min with 1% polyglobin (Talecris Biotherapeutics, Frankfurt am Main, Germany) to prevent unspecific antibody binding. Cells were labelled with the mouse-anti human integrin α7 chain antibody (Abcam) for 20 min. The cells were washed several times using a washing buffer consisting of DPBS containing 0.1% bovine serum albumin (Sigma-Aldrich) and 0.05% sodium azide. Then a phycoerythrin (PE)-conjugated anti-mouse-IgG (Dianova, Hamburg, Germany) was applied for 20 min. The labeled cells were analyzed using FACScan flow cytometer and FACScan Research software (BD Pharmingen) as well as FlowJo (Tree Star, Inc., Ashland, USA).

### Cell proliferation assay

For analyzing the influence of recombinant laminin isoforms on the proliferation of MSCs, 1x10^4^ cells/ml were incubated in 96-well-plates in GMP+ medium or in myogenic differentiation medium with or without adding soluble recombinant laminin isoforms [10 μg/ml each] for seven days at 37°C and 5% CO_2_. The cells were then stained using 0.5% crystal violet solution (Sigma-Aldrich) containing 20% methanol for 30 min. The supernatant was discarded and the fixed cells were washed with tap water. After drying, by adding 100 μl methanol / well, crystal violet was dissolved and the absorption was measured at 595 nm using a fluorescence reader (GENios; Tecan, Crailsheim, Germany). All samples were analyzed in duplicates.

### Cell adhesion assay

Analysis of the adhesive interactions with MSCs, myogenically differentiated MSCs or the two smooth muscle cell types to the different laminin isoforms was carried out as described previously [28]. Briefly, 1 μl of each individual laminin isoform was spotted onto a plastic dish and immobilized by air-drying at room temperature. Nonspecific binding of the different cell types to the plastic dish was prevented by pre-incubation with 1% bovine serum albumin. Then, the individual cell types were allowed to attach for 30 min in serum-free medium supplemented with Mn^2+^, Ca^2+^ and Mg^2+^. By gently rinsing the dish with pre-warmed DPBS non-attached cells could be removed. Specific cell adhesion was evaluated under a Zeiss Axiovert microscope.

For the analysis of the receptor(s) involved in the observed adhesive interactions the different cell types were pre-incubated with function-blocking antibodies against different integrin chains (clone P1B5 for the α3 chain, clone GoH3 for the α6 chain, clone 4B4 for the β1 chain) for 30 min. The adhesion assays were then performed, as described, in the presence of the antibodies.

### Atomic force microscopy

For single cell force measurement by atomic force microscopy with CellHesion^®^ 200 (JPK Instruments, Berlin, Germany), the petri dishes were spotted with recombinant laminin isoforms [0.05 μg/μl] and blocked with bovine serum albumin as described above. TL-2 cantilevers (0.03 N/m nominal spring constant; NanoWorld, Neuchâtel, Switzerland) were coated with Cell Tack (BD Pharmingen) at a 1:30 dilution in 0.1 M sodium bicarbonate for 30 min at room temperature. The cantilever was calibrated on the retract curve and its spring constant was determined using the thermal noise method in the software (JPK Instruments). Single cells were captured by a maximum force of 2 nN during a contact time of 30 sec. Measurements were performed in Leibovitz’s L-15 medium (Life Technologies; supplemented with Mn^2+^, Ca^2+^ and Mg^2+^ ions) at 37°C with a maximum force of 0.5 nN and a contact time of 10 sec on triplicate samples on 3 different spots for n = 9 cells. For cell capture and detachment force measurements, an extend speed of 5 μm/second was used. Force curves were processed with the data processing software (JPK Instruments).

For elasticity measurements, cells were seeded in expansion medium (‘GMP+ medium’) in single petri dishes for each time point and the different media, respectively. For the different time points the medium was exchanged after overnight incubation. Elasticity measurements were performed in L-15 medium using a SiNi tip-cantilever (0.027 N/m nominal spring constant; Budget Sensors, Sofia, Bulgaria) by atomic force microscopy. For determining the spring constant, the cantilever was calibrated on the extend curve and the thermal noise method in the software (JPK Instruments) was used. Single cells without cell-cell contacts were measured over the nucleus with a maximum force of 1 nN, and an extended speed of 5 μm/second in duplicate samples for n = 30 cells. The Young's modulus was calculated using the Hertz-model in the data processing software (JPK Instruments).

### Statistical analysis

All values are expressed as standard error of the mean. By one-way ANOVA analysis or t-test analysis using GraphPad Prism 5 software statistical differences were determined. For *p< 0.05; **p< 0.01; ***p< 0.001, differences were considered to be significant.

## Results

### Laminin chain expression in the urinary sphincter tissue

**Fig 1:**
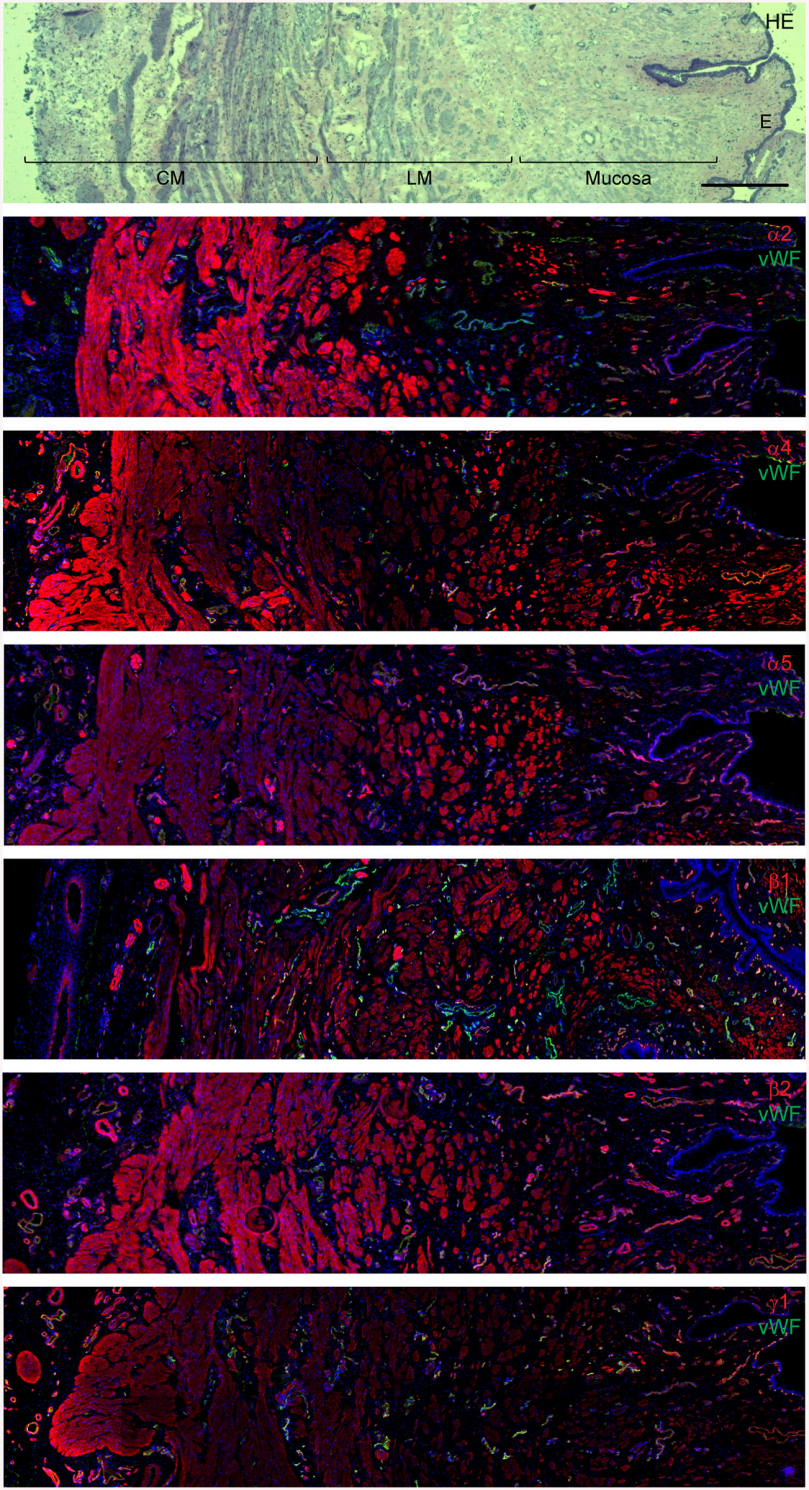
Expression of laminin chains in the mini-pig sphincter tissue. Cryostat sections of an isolated mini-pig sphincter tissue were either stained with hematoxylin-eosin (HE) or labeled with laminin chain specific antibodies (red) together with an antiserum against von Willebrand factor (vWF; green) labeling endothelial cells. By HE staining the inner epithelial lining (E), mucosa, longitudinal smooth muscle layer (LM) and circular smooth muscle layer (CM) can be distinguished. The laminin α chains (α2, α4, α5), β chains (β1, β2) and the γ1 chain are expressed in the mucosa, in the different muscular layers and around endothelial cells, albeit with different intensities. In the merged pictures the weaker expression of the laminin α2, β1 and γ1 chains in some endothelial cells is hidden behind the strong signal for von Willebrand factor staining. Cell nuclei were counterstained in blue with DAPI (bar: 500 μm).

Myogenic tissues can synthesize and secrete several laminin chains (and isoforms) including the α2, α4, α5, β1, β2 and γ1 chains [21, 29–31]. To determine which laminin chains are expressed in the urethral sphincter tissue, cryostat sections of mini-pig sphincter tissues were labelled with laminin-specific antibodies. The specificity of the individual antibodies was shown by Western blotting using different human recombinant laminin isoforms (S1 Fig). The cross-reactivity of these anti-laminin antibodies with mini-pig tissues was also shown by Western blotting (S2 Fig). By hematoxylin/eosin staining the different muscular layers in the sphincter tissue were easily detected: the lumen is lined by an epithelial sheet, followed by a mucosal layer and the longitudinal and circular smooth muscle cell layers of the lissosphincter (Fig 1). Staining for the different laminin chains was observed in the mucosa, in both smooth muscle layers and in the basement membranes of endothelial cells, albeit at different intensities (Fig 1). The most prominent staining for the laminin α2 chain was found in the smooth muscle cell layers, whereas the α4 chain was mainly found in the mucosa and the circular smooth muscle cell layer. The most prominent expression of the laminin α5 chain was observed in the longitudinally oriented smooth muscle cell layer. The laminin β1, β2 and γ1 chains were more or less ubiquitously distributed in the sphincter tissue, albeit the intensities of the staining signals differed (Fig 1). Double staining with von Willebrand factor revealed differences in the composition of the basement membranes of endothelial cells in the sphincter tissue (Fig 1).

### Laminin chain expression in undifferentiated and differentiated MSCs

**Fig 2:**
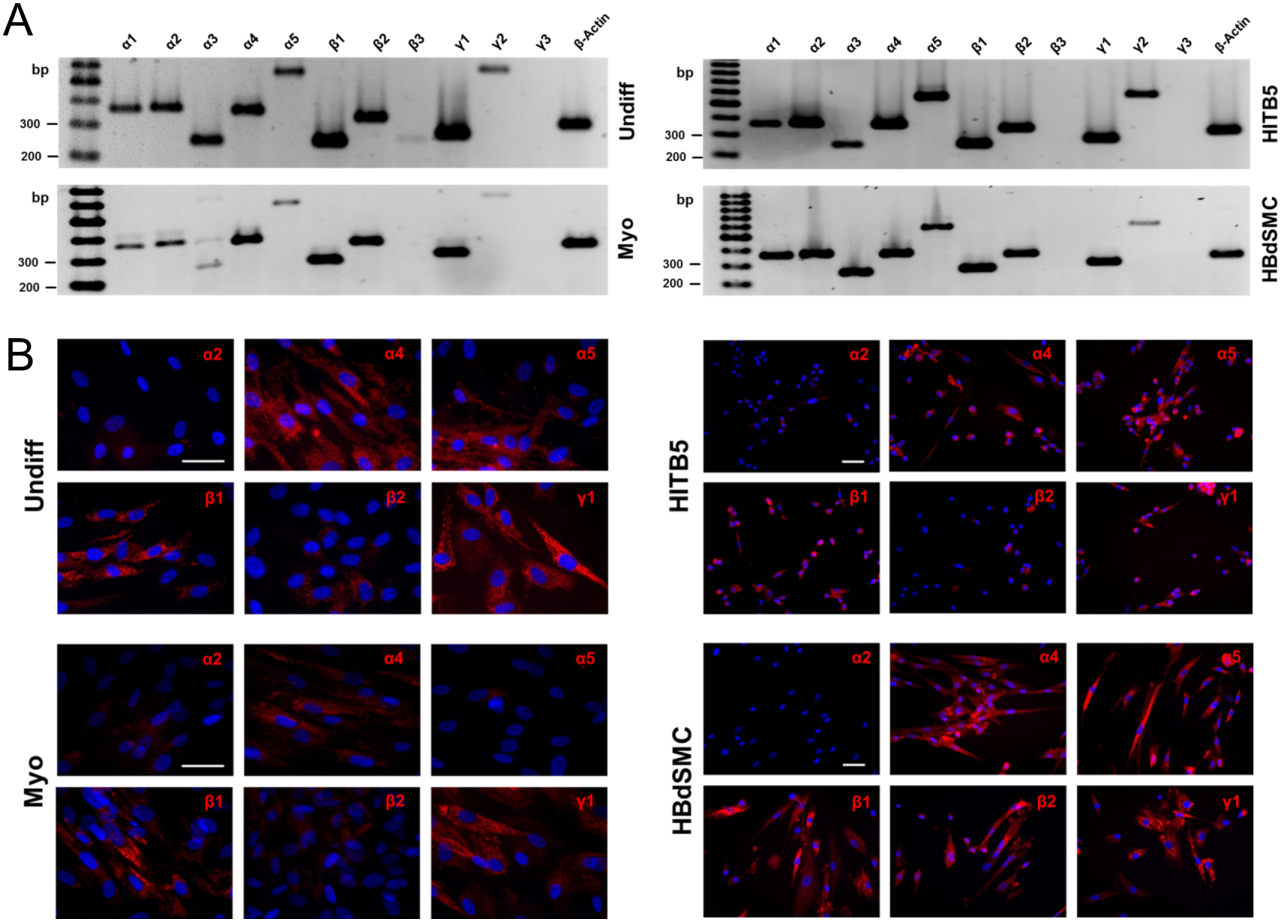
Laminin expression by primary MSCs, the cell line HITB5 and tissue-derived HBdSMC. RT-PCR analyses (A) and immunofluorescence staining (B) of undifferentiated MSCs (Undiff), myogenically differentiated MSCs (Myo), the cell line HITB5 and HBdSMC in early passages suggested the expression of several laminin isoforms. The highest expression was observed for the α4, α5, β1 and γ1 chains. The α2 chain was only weakly expressed. Cell nuclei were counterstained in blue with DAPI (bars: 50 μm).

The expression pattern of the eleven different laminin chains in undifferentiated and myogenically differentiated MSCs was studied by RT-PCR analysis. As positive controls for myogenic cells, the smooth muscle cell line HITB5 and primary human bladder-derived smooth muscle cells (HBdSMC) were also investigated (Fig 2). With the exception of the laminin β3 and γ3 chains, the mRNA of all other laminin chains was found to be expressed by all four cell types studied (Fig 2). By immunofluorescence staining with laminin chain-specific antibodies, a prominent expression of the laminin α4 chain was detected in the undifferentiated and the myogenically differentiated MSCs, whereas the α5 chain was only found in the undifferentiated MSCs, but hardly in the differentiated ones (Fig 2). Accordingly, when analyzing conditioned cell culture supernatants by immunoprecipitation and subsequent Western blotting, prominent signals for the α5 chain were only found in the undifferentiated MSCs, but not in the differentiated cells (S3 Fig). Astonishingly, myogenic differentiation of MSCs did not induce the expression of the laminin α2 chain, the most prominent laminin chain found in myogenic tissues; HBdSMC in culture and the HITB5 smooth muscle cells also showed only weak signals of this laminin chain. As expected, the laminin β1 and γ1 chains were expressed by all cell types analyzed (Fig 2).

### Influence of laminin isoforms on myogenic differentiation of MSCs

**Fig 3:**
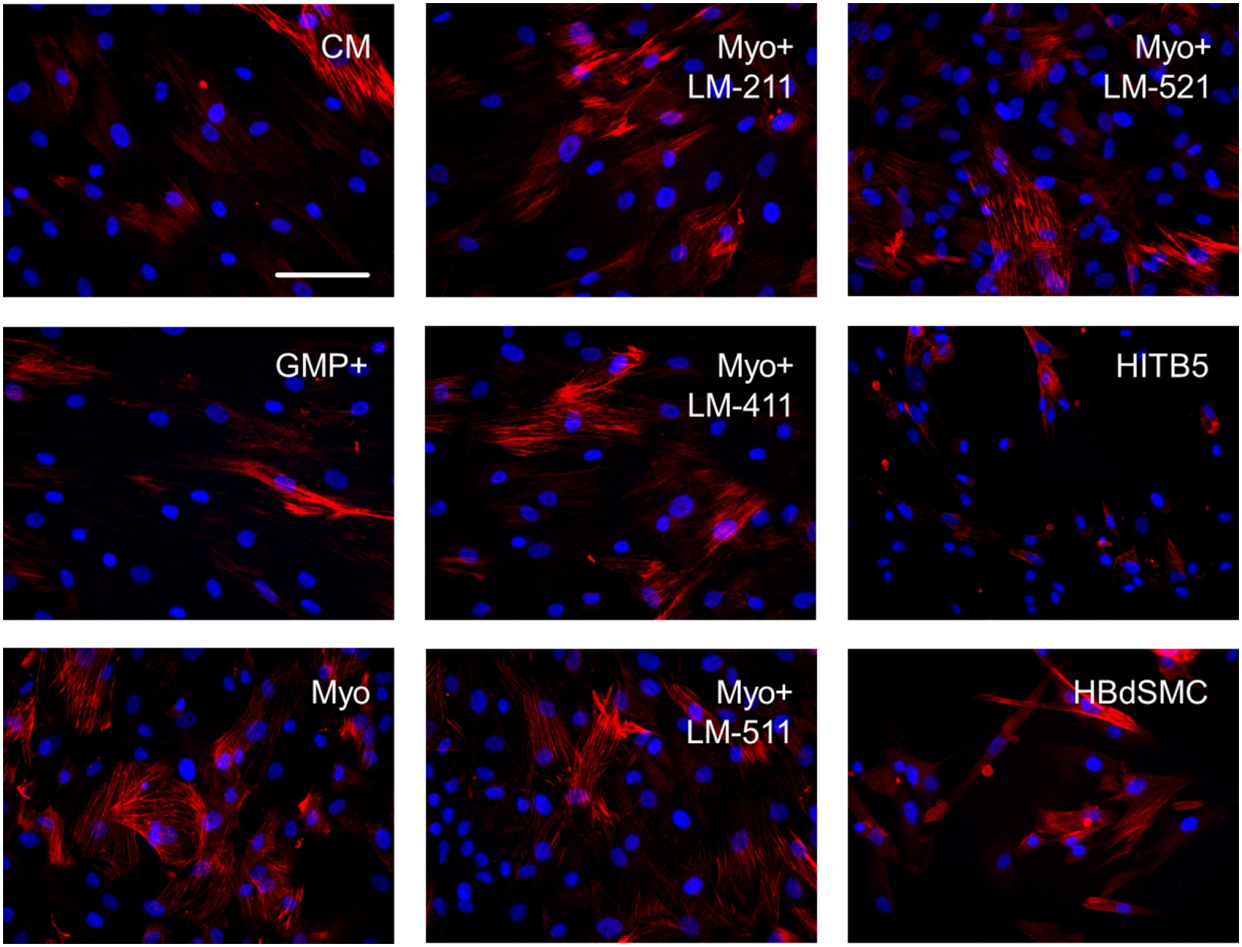
Expression of the myogenic marker molecule calponin by undifferentiated MSCs, MSCs after seven days of myogenic induction, HITB5 cells and HBdSMC. Immunofluorescence staining for calponin indicated that the expression of this intermediate differentiation marker was induced by the myogenic differentiation medium (Myo) compared to the control (CM) or expansion (GMP+) medium, but more or less independent of the treatment with recombinant laminin isoforms LM-211, LM-411, LM-511, LM-521 [10 μg/ml each]. Staining of HITB5 and HBdSMC served as positive controls. Cell nuclei were counterstained in blue with DAPI (bar: 50 μm).

**Fig 4:**
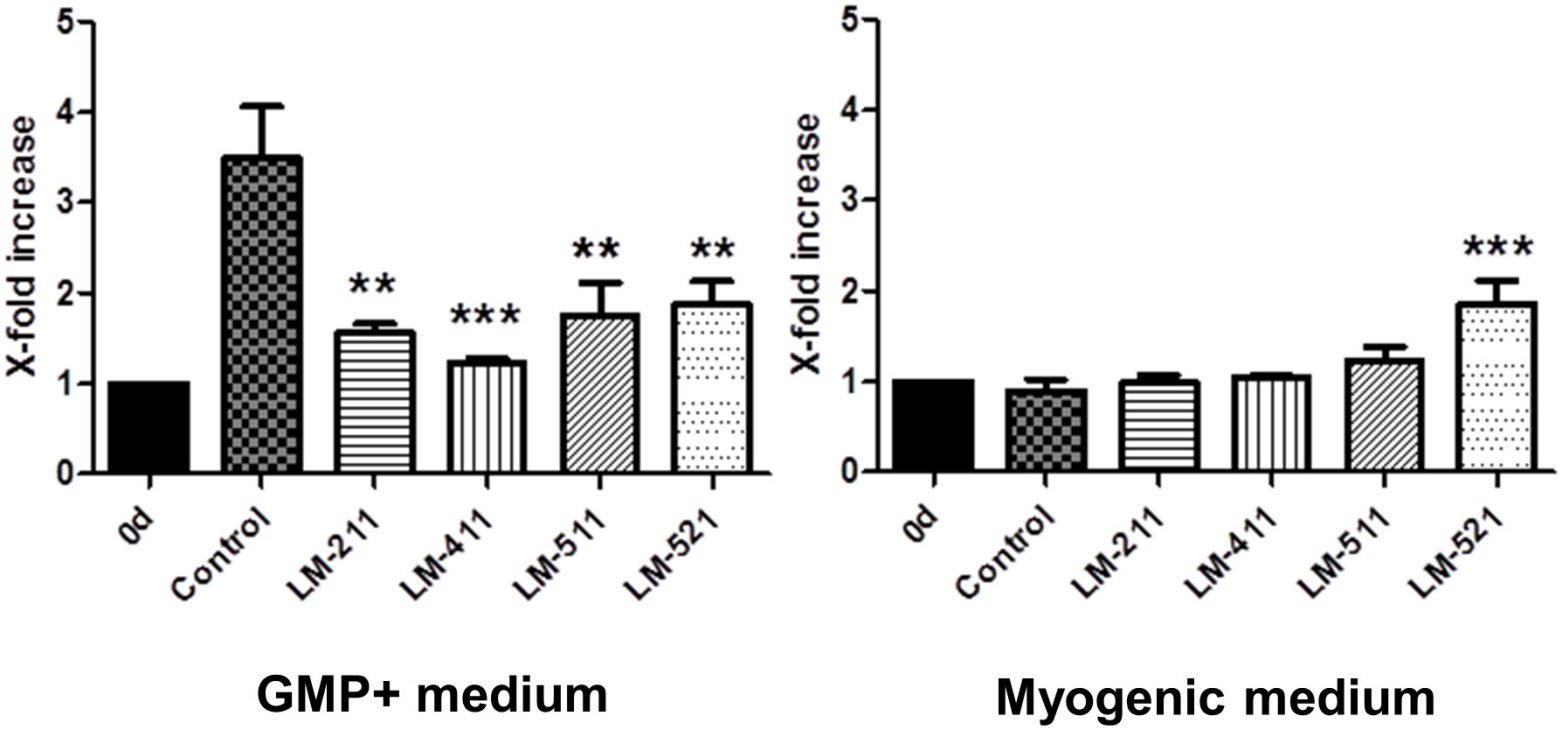
Influence of recombinant laminin isoforms on the proliferation rate of MSCs. MSCs in early passages were grown for seven days in expansion medium (GMP+**)** with or without 10 μg/ml of the recombinant laminin isoforms LM-211, LM-411, LM-511 and LM-521. All laminin isoforms decreased the proliferation rate of MSCs. In contrast, cultivation of MSCs in myogenic differentiation medium with the different recombinant laminin isoforms had no significant effect on the proliferation rate with LM-521 being the exception (n = 3 donors; experiments performed in duplicates; error bars indicate standard error of the mean; one-way ANOVA analysis; **p<0.01; ***p<0.001 in comparison to control).

MSCs cultured for seven days in a medium containing ascorbic acid, TGF-ß1 and PDGF-AB started to differentiate into the myogenic lineage as shown in qRT-PCR analysis and Western blots by an enhanced expression of the myogenic marker molecules αSMA, calponin and transgelin (S4 Fig). An increased expression pattern of calponin was also seen in immunofluorescence staining of myogenically differentiating MSCs compared to MSCs grown in control or expansion medium (Fig 3). However, despite the known influence of laminin isoforms on smooth muscle cell differentiation [22], addition of the human recombinant laminin isoform LM-211 or LM-411, LM-511 and LM-521 isoforms did not apparently alter the expression of calponin in the myogenically differentiating cells (Fig 3). Myogenic differentiation of MSCs inhibited the proliferation of these cells (Fig 4), and LM-211, LM-411 and LM-511 had no influence on the proliferation rate of the myogenically differentiating cells. The LM-521 isoform, however, which is strongly expressed in the longitudinally oriented smooth muscular layer of the sphincter tissue (Fig 1), significantly increased the proliferation rate of the myogenically differentiating MSCs during the seven day culture period (Fig 4). When MSCs were cultured in expansion medium and were not myogenically induced, all four laminin isoforms showed a significant inhibitory effect on MSCs proliferation (Fig 4).

It was recently reported that MSCs treated for 24 h with TGF-ß and PDGF drastically stiffened meaning their elastic moduli increased [32]. We thus compared the elastic moduli of undifferentiated and myogenically differentiated MSCs during a seven day culture period by atomic force microscopy. Although the direct comparison showed that the differentiated MSCs were stiffer than their undifferentiated counterparts, an increase in the elastic moduli was not observed in the differentiated MSCs (S5 Fig). In contrast, MSCs expanded for seven days in culture without differentiation got softer.

### Myogenic differentiation of MSCs diminishes the interaction with laminin isoforms

**Fig 5:**
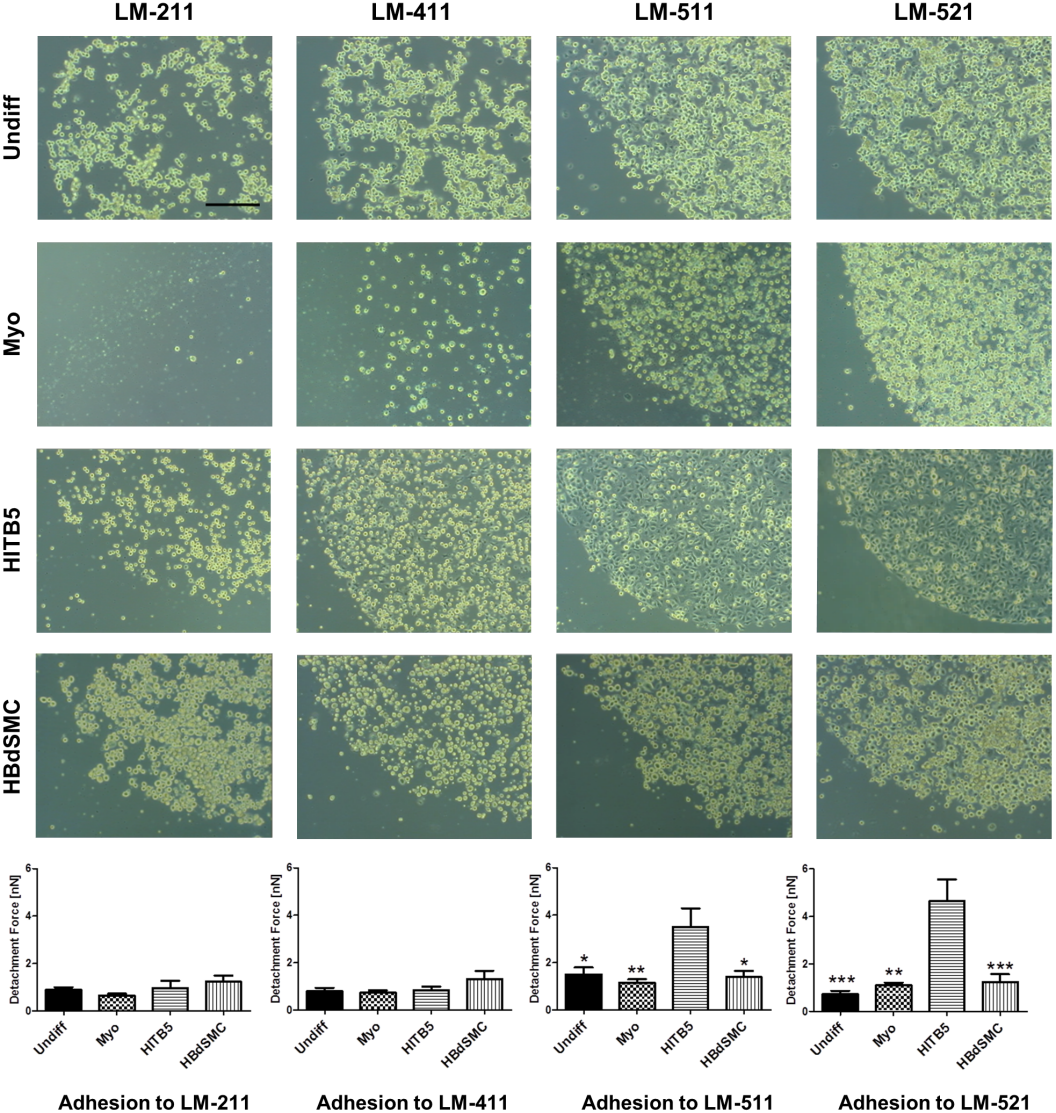
Cell adhesion to plastic-immobilized laminin isoforms. Cell-matrix interactions with undifferentiated MSCs (Undiff), myogenically differentiated MSCs (Myo), the cell line HITB5 and HBdSMC were quantitatively determined by single cell force measurement (n = 9 cells) and qualitatively by spotting assays on different recombinant laminin isoforms. LM-511 and LM-521 were the strongest adhesive substrates for smooth muscle cells and undifferentiated MSCs. Myogenic differentiation diminished the binding of MSCs to LM-411. LM-211 was only a weakly-adhesive substrate for smooth muscle cells and MSCs, and upon myogenic differentiation the adhesive capacity of LM-211 was further diminished (bar: 200 μm; error bars indicate standard error of the mean; one-way ANOVA analysis; *p<0.05; **p<0.01; ***p<0.001 in comparison to HITB5).

By measuring the strength of adhesive interactions by atomic force microscopy on a single cell level with the different laminin isoforms, no obvious differences between the undifferentiated and the myogenically differentiated MSCs were observed (Fig 5). A very strong interaction was detected by atomic force microscopy for the control cell line HITB5 on LM-511 and LM-521, and this strong interaction coincided with a rapid spreading of this cell type on the substrates (Fig 5). A conventional cell adhesion assay with plastic-immobilized laminin isoforms revealed that the myogenic differentiation of MSCs diminished the interaction(s), especially with LM-211, but also with LM-411. The weakest adhesive interactions for all myogenic cell types was found with LM-211, followed by LM-411. The strongest interactions were observed with the laminin isoforms containing an α5 chain (Fig 5).

The expression pattern of the four major laminin receptors of the integrin family, α3β1, α6β1, α6β4 and α7β1 [33] were analyzed on undifferentiated and differentiated MSCs and the two smooth muscle cell types by RT-PCR analysis and by immunofluorescence staining or FACS analysis. Some minor differences were observed for the integrins α3β1 and α6β1 by RT-PCR analysis, but this difference was not seen on the protein level with immunofluorescence staining (S6 Fig). The integrin α6β4 was not expressed by the analyzed cell types (S6 Fig), whereas the integrin α7β1 was strongly expressed by myogenically differentiated MSCs, but not by the smooth muscle cell lines (S7 Fig). Using function-blocking antibodies against the human α3, α6 and β1 integrin chains (a function-blocking antibody against the human integrin α7 chain does not exist so far), an inhibition of adhesive interactions was observed for undifferentiated and differentiated MSCs with the antibody against the β1 integrin chain only with the laminin isoforms LM-211 and LM-411. Adhesive interactions of HITB5 and HBdSMC with the different laminin isoforms could be blocked by anti-α6 or β1 integrin chain antibodies (data not shown).

## Discussion

Bone marrow-derived MSCs are a very interesting option in regenerative medicine of damaged smooth muscle tissues since these cells are highly proliferative *in vitro* and capable of myogenic differentiation. Here we analyzed whether individual laminin isoforms could facilitate the myogenic differentiation process of MSCs into smooth muscle cells. A major laminin isoform of adult muscle tissues is LM-211, but this isoform was hardly expressed by MSCs, not even after *in vitro* myogenic differentiation. MSCs mainly expressed isoforms containing the laminin α4 or α5 chains, and the strongest adhesive interactions were found with LM-511 and LM-521. Laminin isoforms containing an α5 chain, which are the major human laminin isoforms during *in vivo* myogenesis [21], were found to be strongly expressed in the longitudinal smooth muscle layer of the sphincter. In addition, LM-521 is the only laminin isoform capable of enhancing the proliferation of myogenically differentiating MSCs. These results suggest that LM-521 could be successfully applied to improve differentiation protocols of MSCs into smooth muscle cells.

Since isolated human sphincter tissue was not accessible, we analyzed the laminin chain expression pattern in sphincter tissues of mini-pigs which, due to anatomical similarities, serve as a large animal model for sphincter regeneration [34]. In agreement with other studies [25, 26] the anti- laminin antibodies used in this study were shown to be chain-specific and cross-reactive with mini-pig tissues. According to the immunofluorescence data, the strongest signals in the circular and longitudinal smooth muscle cell layers were found for the laminin α2, α5 and β2 chains, identifying LM-221 and LM-521 as the major isoforms in this tissue. Whether this expression pattern can also be found in human tissues will be analyzed as soon as isolated human sphincter tissue will become available.

The MSCs, either undifferentiated or myogenically differentiated, showed a different expression pattern. Although RT-PCR analysis suggested a ubiquitous expression of the laminin alpha chains, the α1 (data not shown) and α2 chains were not or were hardly translated, respectively, into detectable proteins. Laminin isoforms containing the α4 and α5 chains were expressed by undifferentiated MSCs, and this expression pattern was also found in the myogenic control cells. However, during myogenic differentiation of MSCs the expression of the α5 chain-containing isoforms was decreased as shown both by immunoprecipitation and immunofluorescence staining. LM-411, which is a major laminin isoform of smooth muscle cells [35], was the most prominent isoform of *in vitro* myogenically differentiated MSCs. Interestingly, a primary smooth muscle cell isolated from bladder tissue also did not express the laminin α2 chain suggesting that this laminin chain is not prominently expressed by *in vitro* cultured myogenic cells.

For the smooth muscle cell differentiation pathway of MSCs a combination of TGF-ß1, PDGF-AB and ascorbic acid was applied [24, 36, 37]. Here a controlled up-regulation of the myogenic marker molecules αSMA, calponin and transgelin [38, 39] was observed. The GMP+ expansion medium of MSCs contains platelet extract with an undefined concentration of PDGF and TGF-ß which might explain the presence of calponin^+^ cells in MSCs cultures even without induction by the myogenic differentiation medium. A controlled pre-differentiation of the MSCs is considered to be advantageous in a therapeutic application of MSCs for *in vivo* muscle tissue regeneration, since bone marrow-derived undifferentiated MSCs, due to their intrinsic differentiation potential, bear the risk of undesirable osteogenic differentiation [3]. However, a pre-differentiation of MSCs should not drastically alter the biomechanical properties of the cells. Recently it was reported that treatment of murine MSCs with TGF-ß1 alone resulted in a strong stiffening of these cells [32]. Yet, by using human MSCs in the TGF-ß1 containing myogenic medium, we did not observe a significant alteration in the Young’s modulus of these cells, although these cells were stiffer than MSCs cultured in parallel only in expansion medium.

No obvious improvement (e.g., an enhanced number of calponin+ cells) could be detected when MSCs were cultured in myogenic differentiation medium together with the different recombinant laminin isoforms. This was in sharp contrast to the successful differentiation of cardiomyocytes from human induced pluripotent stem cells where the laminin isoform LM-521 in a chemically defined medium was determined as the optimal matrix [40]. Differentiation of intestinal smooth muscle cells was also dependent on laminin α5 chain containing isoforms [29]. Whether the different applications (10 μg/ml soluble laminins in our study vs. 2.5 μg/cm^2^ laminin coating in [40]) could explain the different outcomes has still to be determined.

Although LM-521 could not enhance the number of myogenically differentiating calponin+ cells, it was the only laminin isoform that could induce a proliferation of MSCs in the myogenic differentiation medium. This isoform is also suitable to expand human embryonic stem cells in a chemically defined medium under xeno-free conditions [41]. The proliferation of undifferentiated MSCs, however, was significantly inhibited by all laminin isoforms tested, including LM-521.

Responses to the different laminin isoforms are mediated by membrane-bound receptors, mainly of the integrin family. The major receptors for the laminin α4 and α5 chain-containing isoforms are the integrins α3β1, α6β1, α7β1 and α6β4 [33], and all of these integrins, except α6β4, can also interact with the laminin α2 chain [30]. The α3β1 and the α6β1 integrins, but not the α6β4 integrin, were also shown to be strongly expressed by undifferentiated and by myogenically differentiating MSCs. An up-regulation of the integrin α7β1 could even be observed for the myogenically differentiating MSCs. Nevertheless, myogenic differentiation of the MSCs decreased their adhesion to the laminin isoforms, especially to the isoform containing the α2 or α4 chains. Adhesion to the α5 chain-containing isoforms was largely unaffected. Since function-blocking antibodies against the integrin α3 and α6 chains also did not alter the adhesion of both undifferentiated and differentiated MSCs, the integrin expression pattern does not give a plausible clue for the observed adhesive interactions of those cells. Whether other laminin receptors such as Lutheran or α-dystroglycan can explain the different adhesive characteristics is still unresolved [31, 42].

We hypothesized that differences in the observed adhesive interactions should be the result of different adhesive strengths, measurable by atomic force microscopy. However, quantification of the adhesive forces of singular MSCs to the different laminin isoforms did not reveal significant differences between the undifferentiated and differentiated MSCs, although the number of experiments was much higher than the semi-quantitative spot adhesion assay. Only the myogenic HITB5 cells adhered much more strongly to LM-511/521, but this phenomenon was also reflected by a rapid cell spreading of HITB5 cells on the laminin spots in the semi-quantitative assay. A similar cell spreading was not observed for all the other cell types analyzed.

Differentiation of MSCs could not only be influenced by biochemical signaling through cytokines, growth factors, or extracellular matrix molecules but also by biophysical parameters such as the elasticity of the substrate, and the geometry or physical strain [43–45]. The identification of LM-521 as a proliferation-inducing and highly adhesive substrate for myogenically differentiating bone marrow-derived MSCs could help design protocols with optimal conditions for the desired differentiation pathway that could ultimately find its way to the clinic.

## Supporting Information

S1 Fig Specificity of anti-laminin chain-specific antibodies. Using the recombinant laminin isoforms LM-211, LM-411, LM-421, LM-511 and LM-521 [200 ng/lane], the laminin chain-specific antibodies against the human laminin α4, α5 and β2 chains and against the mouse laminin ß1 chain exclusively recognized their specific laminin bands in the Western blots.
(TIF)

S2 Fig Determination of species cross-reactivity of anti-laminin chain-specific antibodies. Using lysates from mini-pig bladder tissue, the antibodies against human laminin α4, α5, and β2 chains and the mouse laminin ß1 chain showed specific bands by Western blotting. The recombinant human laminin isoforms LM-411, -511 and -521 [200 ng/lane] were loaded as positive controls. Differences in the molecular weight position for the α4 and α5 chains in the lysates can be explained by proteolytic processing of the laminin chains in the tissue.
(TIF)

S3 Fig Secretion of laminin isoforms by MSCs and smooth muscle cells. Conditioned media of undifferentiated (Undiff) and myogenically differentiated MSCs (Myo), HITB5 and HBdSMC cultured for 48 h were used for immunoprecipitation with antibodies against the human laminin α4 and α5 chains. The precipitated proteins were separated by SDS-PAGE and then analyzed by Western blotting with antibodies against the laminin β1 chain. An enhanced secretion of the laminin α5 chain could be seen for undifferentiated MSCs and the HITB5 cell line, whereas a weak secretion of the laminin α4 chain was only found in undifferentiated MSCs. As positive controls, the recombinant laminin isoforms LM-411 and LM-511 were used [200 ng/lane].
(TIF)

S4 Fig Expression of the myogenic marker molecules αSMA, calponin and transgelin before and after myogenic differentiation. MSCs were analyzed for αSMA (A), calponin (B) and transgelin (C) expression at day 0 and day 7 of myogenic differentiation by qRT-PCR and Western blotting. Myogenically differentiated cells expressed significantly higher amounts of calponin and transgelin compared to MSCs cultured for seven days in control medium or to MSCs at day 0. A tendency towards a higher αSMA-expression could be detected at the transcriptional level. (n = 5 donors; error bars indicate standard error of the mean, one-way ANOVA analysis; *p<0.05 in comparison to day 0). For the different Western blots, vinculin labeling was used as a loading control.
(TIF)

S5 Fig Evaluation of the elasticity of MSCs cultured in different media. Young’s modulus as a measure of the stiffness of the cells was determined for MSCs cultured in expansion media (GMP+). During the seven days of culture these cells became softer, in contrast to MSCs cultured in myogenic differentiation medium. For comparison the elasticities of HBdSMC and HITB5 were determined. (n = 3 donors; error bars indicate standard error of the mean; one-way ANOVA analysis; *p<0.05; ***p<0.001).
(TIF)

S6 Fig Expression of laminin binding integrin receptors on MSCs and smooth muscle cells. RT-PCR analyses and immunofluorescence staining of undifferentiated MSCs (Undiff), myogenically differentiated MSCs (Myo), HITB5 and HBdSMC indicated the expression of several laminin-binding integrin receptors. The integrin-α3 chain (ITGA3), the integrin-α6 chain (ITGA6) and the integrin-β1 chain (ITGB1) were strongly expressed by all analyzed cell types. The integrin-β4 chain (ITGB4) was not expressed by these cells. Cell nuclei were counterstained in blue with DAPI (bars: 100 μm).
(TIF)

S7 Fig Expression pattern of integrin-α7 (ITGA7) on MSCs and smooth muscle cells. RT-PCR and flow cytometry analysis showed the expression of the integrin-α7 chain on undifferentiated MSCs (Undiff) and myogenically differentiated MSCs (Myo), but not or almost not on HITB5 and HBdSMC. The highest expression was observed for myogenically differentiated MSCs. Undifferentiated MSCs expressed ITGA7 at an intermediate level (n = 3 donors; error bars indicate standard error of the mean; t-test analysis; *p<0.05).
(TIF)

## References

[1] Garcia-Castro J, Trigueros C, Madrenas J, Perez-Simon JA, Rodriguez R, Menendez P. Mesenchymal stem cells and their use as cell replacement therapy and disease modelling tool. J Cell Mol Med. 2008;12(6B):2552–65. 10.1111/j.1582-4934.2008.00516.x 19210755 PMC3828873

[2] Caplan AI. Adult mesenchymal stem cells for tissue engineering versus regenerative medicine. J Cell Physiol. 2007;213(2):341–7. 10.1002/jcp.21200 17620285

[3] Bianco P, Cao X, Frenette PS, Mao JJ, Robey PG, Simmons PJ, et al The meaning, the sense and the significance: translating the science of mesenchymal stem cells into medicine. Nat Med. 2013;19(1):35–42. 10.1038/nm.3028 23296015 PMC3998103

[4] Frenette PS, Pinho S, Lucas D, Scheiermann C. Mesenchymal stem cell: keystone of the hematopoietic stem cell niche and a stepping-stone for regenerative medicine. Annu Rev Immunol. 2013;31:285–316. 10.1146/annurev-immunol-032712-095919 23298209

[5] Kurpinski K, Lam H, Chu J, Wang A, Kim A, Tsay E, et al Transforming growth factor-beta and notch signaling mediate stem cell differentiation into smooth muscle cells. Stem Cells. 2010;28(4):734–42. 10.1002/stem.319 20146266

[6] de la Garza-Rodea AS, van der Velde-van Dijke I, Boersma H, Goncalves MA, van Bekkum DW, de Vries AA, et al Myogenic properties of human mesenchymal stem cells derived from three different sources. Cell Transplant. 2012;21(1):153–73. 10.3727/096368911X580554 21669036

[7] Yu H, Lui YS, Xiong S, Leong WS, Wen F, Nurkahfianto H, et al Insights into the role of focal adhesion modulation in myogenic differentiation of human mesenchymal stem cells. Stem Cells Dev. 2013;22(1):136–47. 10.1089/scd.2012.0160 22765653 PMC3528092

[8] Alimperti S, You H, George T, Agarwal SK, Andreadis ST. Cadherin-11 regulates both mesenchymal stem cell differentiation into smooth muscle cells and the development of contractile function in vivo. J Cell Sci. 2014;127(Pt 12):2627–38. 10.1242/jcs.134833 24741067 PMC4058109

[9] Galli D, Vitale M, Vaccarezza M. Bone marrow-derived mesenchymal cell differentiation toward myogenic lineages: facts and perspectives. Biomed Res Int 2014;2014:762695 10.1155/2014/762695 25054145 PMC4099119

[10] Liu J, Wang Y, Wu Y, Ni B, Liang Z. Sodium butyrate promotes the differentiation of rat bone marrow mesenchymal stem cells to smooth muscle cells through histone acetylation. PLoS One. 2014;9(12):e116183 10.1371/journal.pone.0116183 25548915 PMC4280132

[11] Klein G, Hart ML, Brinchmann JE, Rolauffs B, Stenzl A, Sievert KD, et al Mesenchymal stromal cells for sphincter regeneration. Adv Drug Deliv Rev. 2015;82–83:123–36. 10.1016/j.addr.2014.10.026 25451135

[12] Ulrich C, Rolauffs B, Abele H, Bonin M, Nieselt K, Hart ML, et al Low osteogenic differentiation potential of placenta-derived mesenchymal stromal cells correlates with low expression of the transcription factors Runx2 and Twist2. Stem Cells Dev. 2013;22(21):2859–72. 10.1089/scd.2012.0693 23763516 PMC3804084

[13] Ragni E, Montemurro T, Montelatici E, Lavazza C, Vigano M, Rebulla P, et al Differential microRNA signature of human mesenchymal stem cells from different sources reveals an "environmental-niche memory" for bone marrow stem cells. Exp Cell Res. 2013;319(10):1562–74. 10.1016/j.yexcr.2013.04.002 23578766

[14] Furuta A, Carr LK, Yoshimura N, Chancellor MB. Advances in the understanding of sress urinary incontinence and the promise of stem-cell therapy. Rev Urol. 2007;9(3):106–12. 17934567 PMC2002500

[15] Wallner C, Dabhoiwala NF, DeRuiter MC, Lamers WH. The anatomical components of urinary continence. Eur Urol. 2009;55(4):932–43. 10.1016/j.eururo.2008.08.032 18755535

[16] Hart ML, Neumayer KM, Vaegler M, Daum L, Amend B, Sievert KD, et al Cell-based therapy for the deficient urinary sphincter. Curr Urol Rep. 2013;14(5):476–87. 10.1007/s11934-013-0352-7 23824516

[17] Hohenester E, Yurchenco PD. Laminins in basement membrane assembly. Cell Adh Migr. 2013;7(1):56–63. 10.4161/cam.21831 23076216 PMC3544787

[18] Aumailley M. The laminin family. Cell Adh Migr. 2013;7(1):48–55. 10.4161/cam.22826 23263632 PMC3544786

[19] Domogatskaya A, Rodin S, Tryggvason K. Functional diversity of laminins. Annu Rev Cell Dev Biol. 2012;28:523–53. 10.1146/annurev-cellbio-101011-155750 23057746

[20] Rodin S, Domogatskaya A, Strom S, Hansson EM, Chien KR, Inzunza J, et al Long-term self-renewal of human pluripotent stem cells on human recombinant laminin-511. Nat Biotechnol. 2010;28(6):611–5. 10.1038/nbt.1620 20512123

[21] Gawlik KI, Durbeej M. Skeletal muscle laminin and MDC1A: pathogenesis and treatment strategies. Skelet Muscle. 2011;1(1):9 10.1186/2044-5040-1-9 21798088 PMC3156650

[22] Nguyen NM, Senior RM. Laminin isoforms and lung development: all isoforms are not equal. Dev Biol. 2006;294(2):271–9. 10.1016/j.ydbio.2006.03.032 16643883

[23] Holmberg J, Durbeej M. Laminin-211 in skeletal muscle function. Cell Adh Migr. 2013;7(1):111–21. 10.4161/cam.22618 23154401 PMC3544775

[24] Narita Y, Yamawaki A, Kagami H, Ueda M, Ueda Y. Effects of transforming growth factor-beta 1 and ascorbic acid on differentiation of human bone-marrow-derived mesenchymal stem cells into smooth muscle cell lineage. Cell Tissue Res. 2008;333(3):449–59. 10.1007/s00441-008-0654-0 18607632

[25] Wondimu Z, Geberhiwot T, Ingerpuu S, Juronen E, Xie X, Lindbom L, et al An endothelial laminin isoform, laminin 8 (alpha4beta1gamma1), is secreted by blood neutrophils, promotes neutrophil migration and extravasation, and protects neutrophils from apoptosis. Blood. 2004;104(6):1859–66. 10.1182/blood-2004-01-0396 15172971

[26] Wondimu Z, Omrani S, Ishikawa T, Javed F, Oikawa Y, Virtanen I, et al A novel monoclonal antibody to human laminin alpha5 chain strongly inhibits integrin-mediated cell adhesion and migration on laminins 511 and 521. PLoS One. 2013;8(1):e53648 10.1371/journal.pone.0053648 23308268 PMC3538678

[27] Bustin SA, Benes V, Garson JA, Hellemans J, Huggett J, Kubista M, et al The MIQE guidelines: minimum information for publication of quantitative real-time PCR experiments. Clin Chem. 2009;55(4):611–22. 10.1373/clinchem.2008.112797 19246619

[28] Hergeth SP, Aicher WK, Essl M, Schreiber TD, Sasaki T, Klein G. Characterization and functional analysis of osteoblast-derived fibulins in the human hematopoietic stem cell niche. Exp Hematol. 2008;36(8):1022–34. Epub 2008/05/13. S0301-472X(08)00127-6 [pii] 10.1016/j.exphem.2008.03.013 18468769

[29] Bolcato-Bellemin AL, Lefebvre O, Arnold C, Sorokin L, Miner JH, Kedinger M, et al Laminin alpha5 chain is required for intestinal smooth muscle development. Dev Biol. 2003;260(2):376–90. 12921739 10.1016/s0012-1606(03)00254-9

[30] Schéele S, Nyström A, Durbeej M, Talts J, Ekblom M, Ekblom P. Laminin isoforms in development and disease: Review. J Mol Med (Berl). 2007;85(8):825–36. 17426950 10.1007/s00109-007-0182-5

[31] Yousif LF, Di Russo J, Sorokin L. Laminin isoforms in endothelial and perivascular basement membranes. Cell Adh Migr. 2013;7(1):101–10. 10.4161/cam.22680 23263631 PMC3544773

[32] Ghosh D, Lili L, McGrail DJ, Matyunina LV, McDonald JF, Dawson MR. Integral role of platelet-derived growth factor in mediating transforming growth factor-beta1-dependent mesenchymal stem cell stiffening. Stem Cells Dev. 2014;23(3):245–61. 10.1089/scd.2013.0240 24093435 PMC3904528

[33] Nishiuchi R, Takagi J, Hayashi M, Ido H, Yagi Y, Sanzen N, et al Ligand-binding specificities of laminin-binding integrins: a comprehensive survey of laminin-integrin interactions using recombinant alpha3beta1, alpha6beta1, alpha7beta1 and alpha6beta4 integrins. Matrix Biol. 2006;25(3):189–97. 10.1016/j.matbio.2005.12.001 16413178

[34] Vaegler M, Maerz JK, Amend B, da Silva LA, Mannheim JG, Fuchs K, et al Labelling and tracking of human mesenchymal stromal cells in preclinical studies and large animal models of degenerative diseases. Curr Stem Cell Res Ther. 2014;9(5):444–50. 24853377 10.2174/1574888x09666140521144559

[35] Hallmann R, Horn N, Selg M, Wendler O, Pausch F, Sorokin LM. Expression and function of laminins in the embryonic and mature vasculature. Physiol Rev. 2005;85(3):979–1000. Epub 2005/07/01. 85/3/979 [pii] 10.1152/physrev.00014.2004 15987800

[36] Tian H, Bharadwaj S, Liu Y, Ma H, Ma PX, Atala A, et al Myogenic differentiation of human bone marrow mesenchymal stem cells on a 3D nano fibrous scaffold for bladder tissue engineering. Biomaterials. 2010;31(5):870–7. 10.1016/j.biomaterials.2009.10.001 19853294 PMC2787773

[37] Williams C, Xie AW, Emani S, Yamato M, Okano T, Emani SM, et al A comparison of human smooth muscle and mesenchymal stem cells as potential cell sources for tissue-engineered vascular patches. Tissue Eng Part A. 2012;18(9–10):986–98. 10.1089/ten.TEA.2011.0172 22145703

[38] Beamish JA, He P, Kottke-Marchant K, Marchant RE. Molecular regulation of contractile smooth muscle cell phenotype: implications for vascular tissue engineering. Tissue Eng Part B Rev. 2010;16(5):467–91. 10.1089/ten.TEB.2009.0630 20334504 PMC2943591

[39] Rensen SS, Doevendans PA, van Eys GJ. Regulation and characteristics of vascular smooth muscle cell phenotypic diversity. Neth Heart J. 2007;15(3):100–8. 17612668 10.1007/BF03085963 PMC1847757

[40] Burridge PW, Matsa E, Shukla P, Lin ZC, Churko JM, Ebert AD, et al Chemically defined generation of human cardiomyocytes. Nat Methods. 2014;11(8):855–60. 10.1038/nmeth.2999 24930130 PMC4169698

[41] Rodin S, Antonsson L, Niaudet C, Simonson OE, Salmela E, Hansson EM, et al Clonal culturing of human embryonic stem cells on laminin-521/E-cadherin matrix in defined and xeno-free environment. Nat Commun. 2014;5:3195 10.1038/ncomms4195 24463987

[42] Kikkawa Y, Miner JH. Review: Lutheran/B-CAM: a laminin receptor on red blood cells and in various tissues. Connect Tissue Res. 2005;46(4–5):193–9. 10.1080/03008200500344074 16546822

[43] Engler AJ, Sen S, Sweeney HL, Discher DE. Matrix elasticity directs stem cell lineage specification. Cell. 2006;126(4):677–89. 10.1016/j.cell.2006.06.044 16923388

[44] Nava MM, Raimondi MT, Pietrabissa R. Controlling self-renewal and differentiation of stem cells via mechanical cues. J Biomed Biotechnol. 2012;2012:797410 10.1155/2012/797410 23091358 PMC3471035

[45] Park JS, Chu JS, Tsou AD, Diop R, Tang Z, Wang A, et al The effect of matrix stiffness on the differentiation of mesenchymal stem cells in response to TGF-beta. Biomaterials. 2011;32(16):3921–30. 10.1016/j.biomaterials.2011.02.019 21397942 PMC3073995

