# Supplementary figures and images for: Mesenchymal Stromal Cells for Sphincter Regeneration: Role of Laminin Isoforms upon Myogenic Differentiation
Source: PLoS One. 2015 Sep 25;10(9):e0137419. doi: 10.1371/journal.pone.0137419 (PMC4583377; doi:10.1371/journal.pone.0137419)

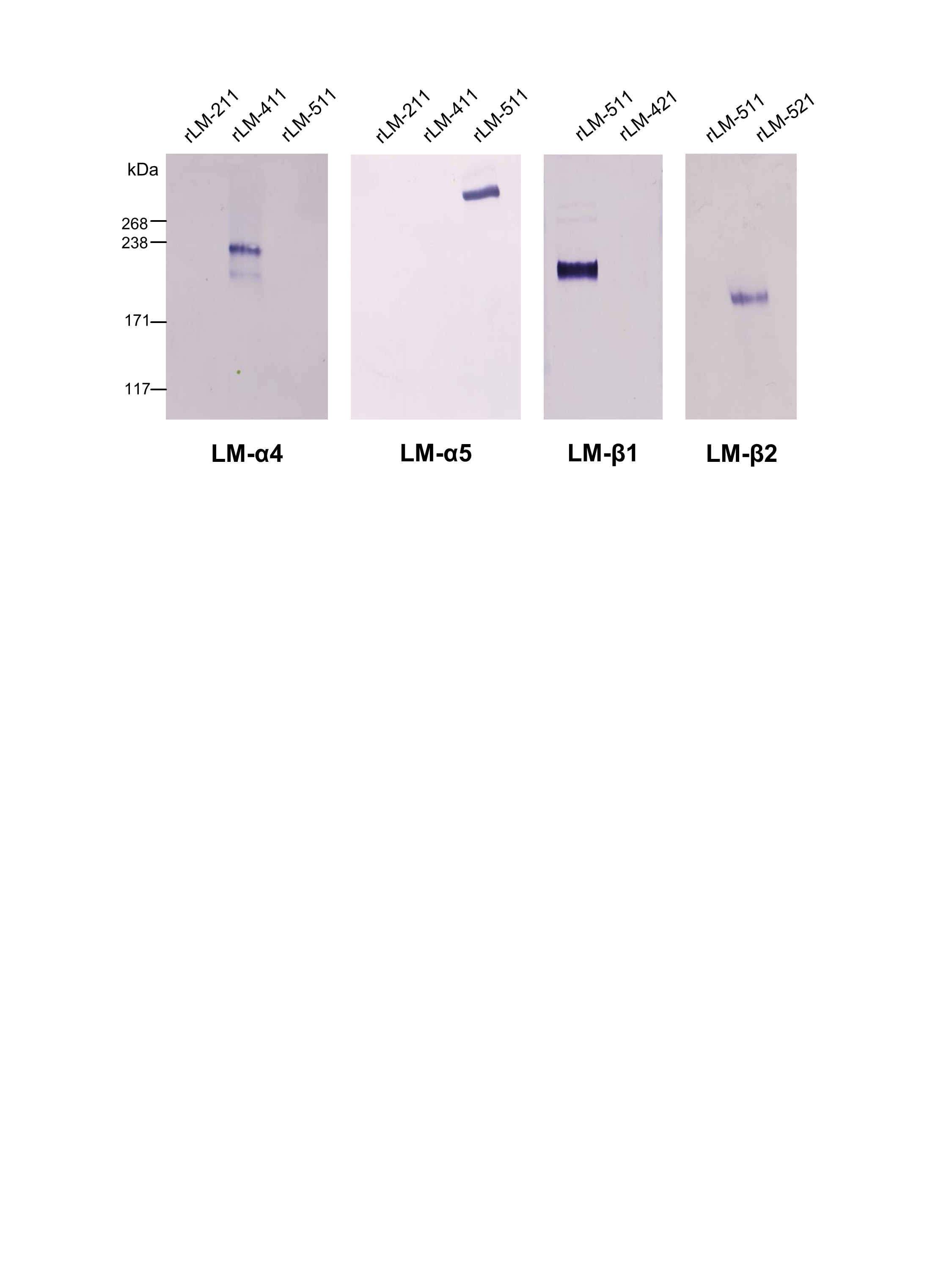

Supplement: S1 Fig — Using the recombinant laminin isoforms LM-211, LM-411, LM-421, LM-511 and LM-521 [200 ng/lane], the laminin chain-specific antibodies against the human laminin α4, α5 and β2 chains and against the mouse laminin ß1 chain exclusively recognized their specific laminin bands in the Western blots. (TIF) [file pone.0137419.s001.tif]

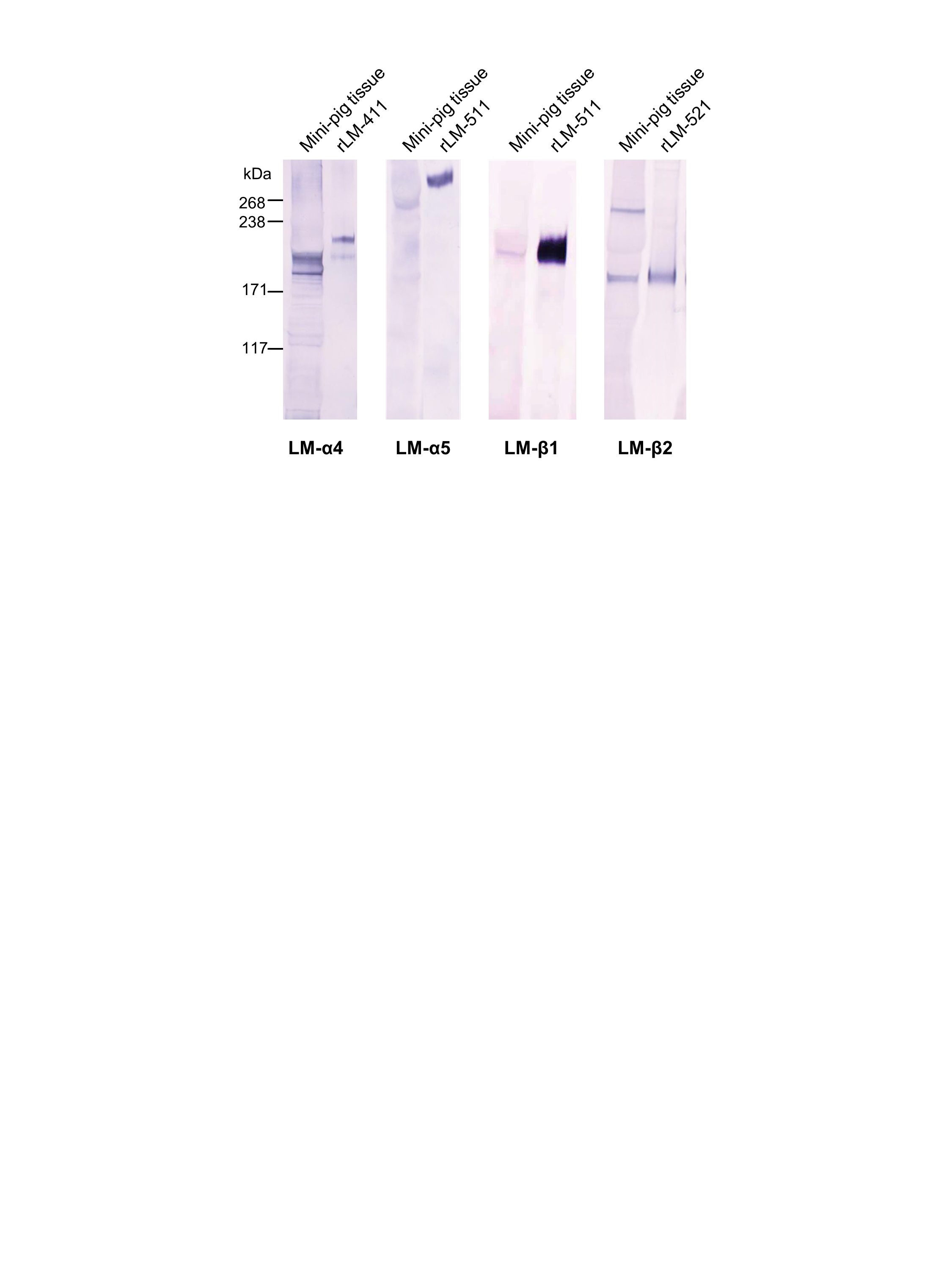

Supplement: S2 Fig — Using lysates from mini-pig bladder tissue, the antibodies against human laminin α4, α5, and β2 chains and the mouse laminin ß1 chain showed specific bands by Western blotting. The recombinant human laminin isoforms LM-411, -511 and -521 [200 ng/lane] were loaded as positive controls. Differences in the molecular weight position for the α4 and α5 chains in the lysates can be explained by proteolytic processing of the laminin chains in the tissue. (TIF) [file pone.0137419.s002.tif]

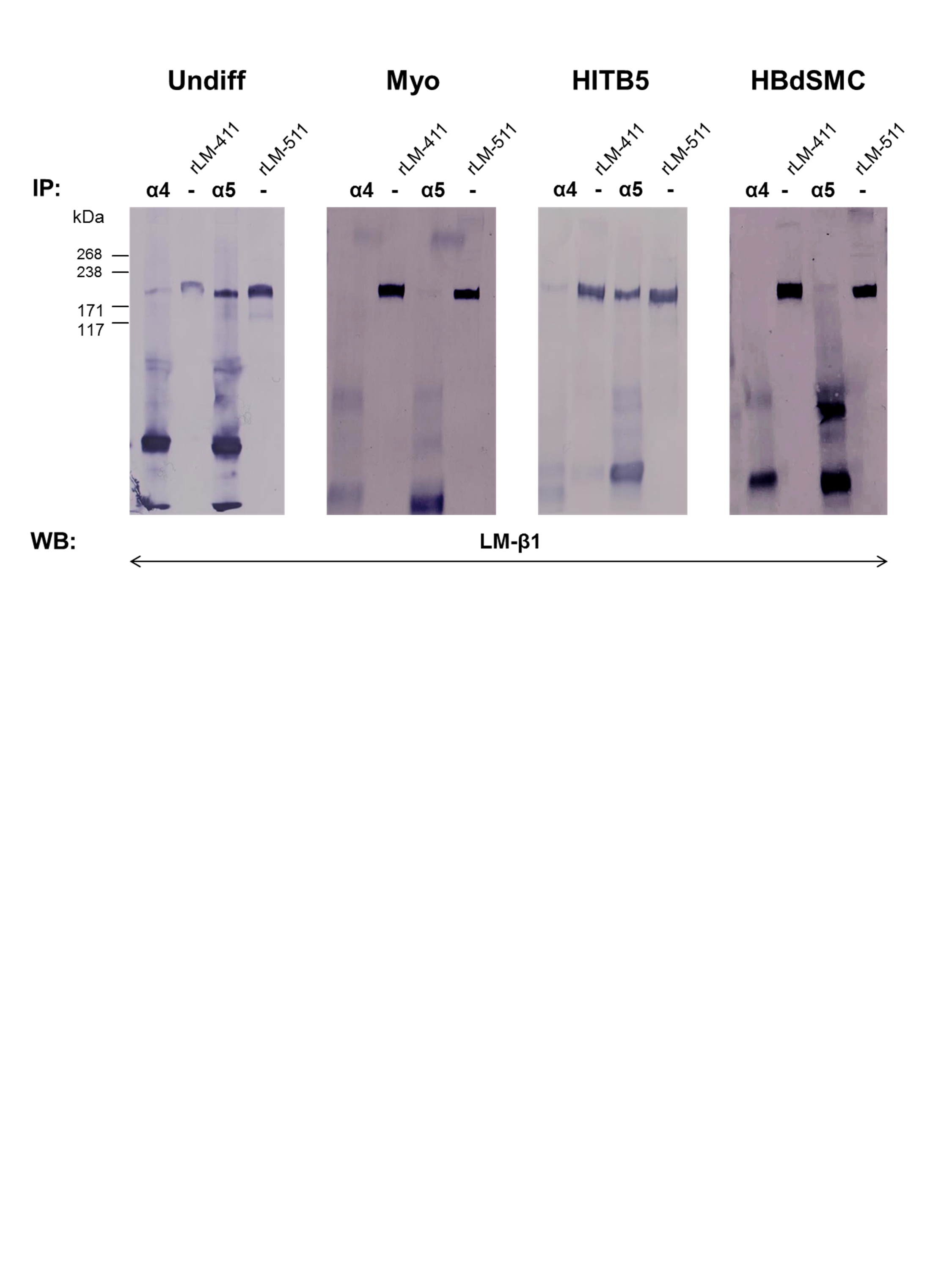

Supplement: S3 Fig — Conditioned media of undifferentiated (Undiff) and myogenically differentiated MSCs (Myo), HITB5 and HBdSMC cultured for 48 h were used for immunoprecipitation with antibodies against the human laminin α4 and α5 chains. The precipitated proteins were separated by SDS-PAGE and then analyzed by Western blotting with antibodies against the laminin β1 chain. An enhanced secretion of the laminin α5 chain could be seen for undifferentiated MSCs and the HITB5 cell line, whereas a weak secretion of the laminin α4 chain was only found in undifferentiated MSCs. As positive controls, the recombinant laminin isoforms LM-411 and LM-511 were used [200 ng/lane]. (TIF) [file pone.0137419.s003.tif]

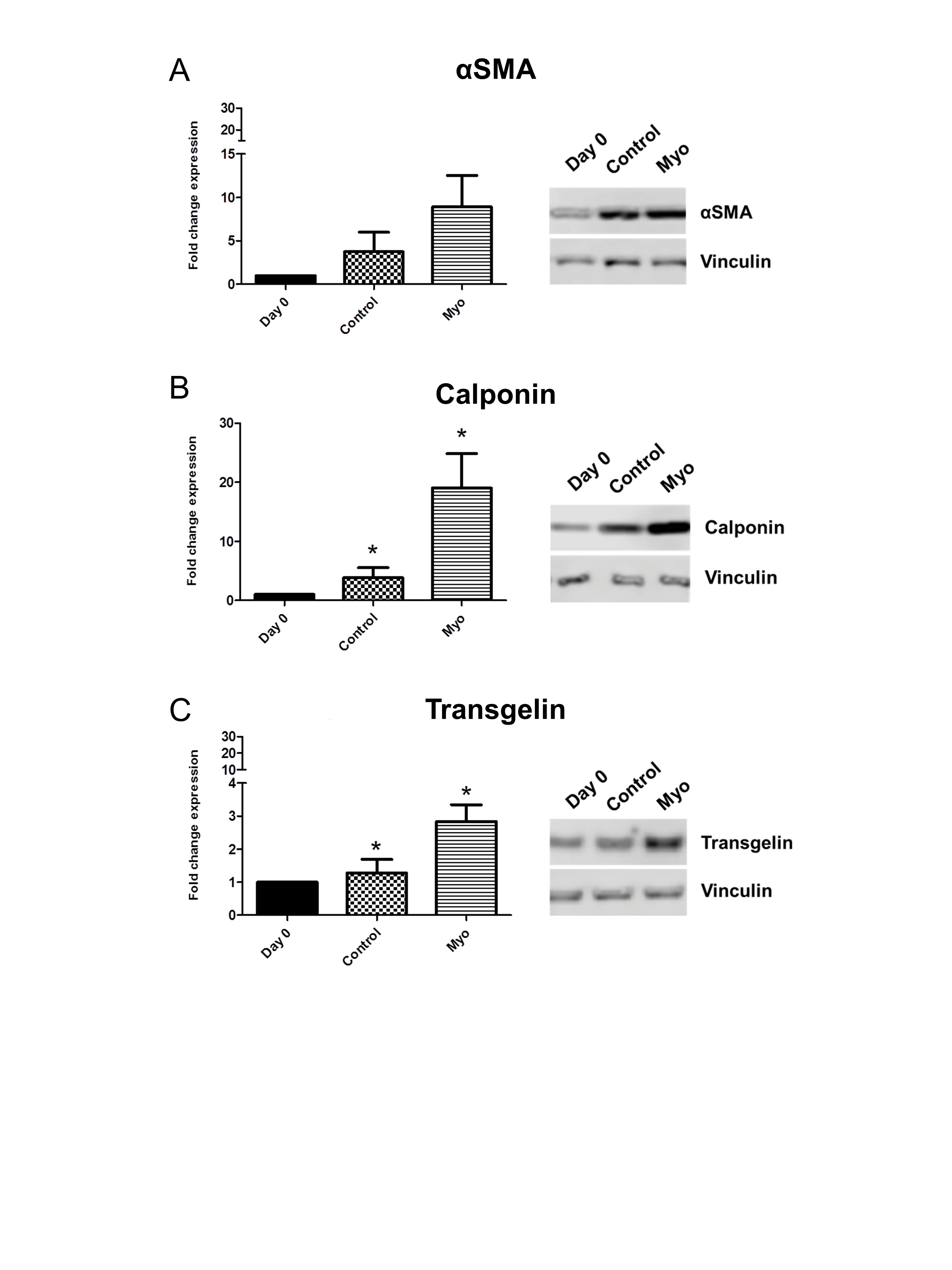

Supplement: S4 Fig — MSCs were analyzed for αSMA (A), calponin (B) and transgelin (C) expression at day 0 and day 7 of myogenic differentiation by qRT-PCR and Western blotting. Myogenically differentiated cells expressed significantly higher amounts of calponin and transgelin compared to MSCs cultured for seven days in control medium or to MSCs at day 0. A tendency towards a higher αSMA-expression could be detected at the transcriptional level. (n = 5 donors; error bars indicate standard error of the mean, one-way ANOVA analysis; *p<0.05 in comparison to day 0). For the different Western blots, vinculin labeling was used as a loading control. (TIF) [file pone.0137419.s004.tif]

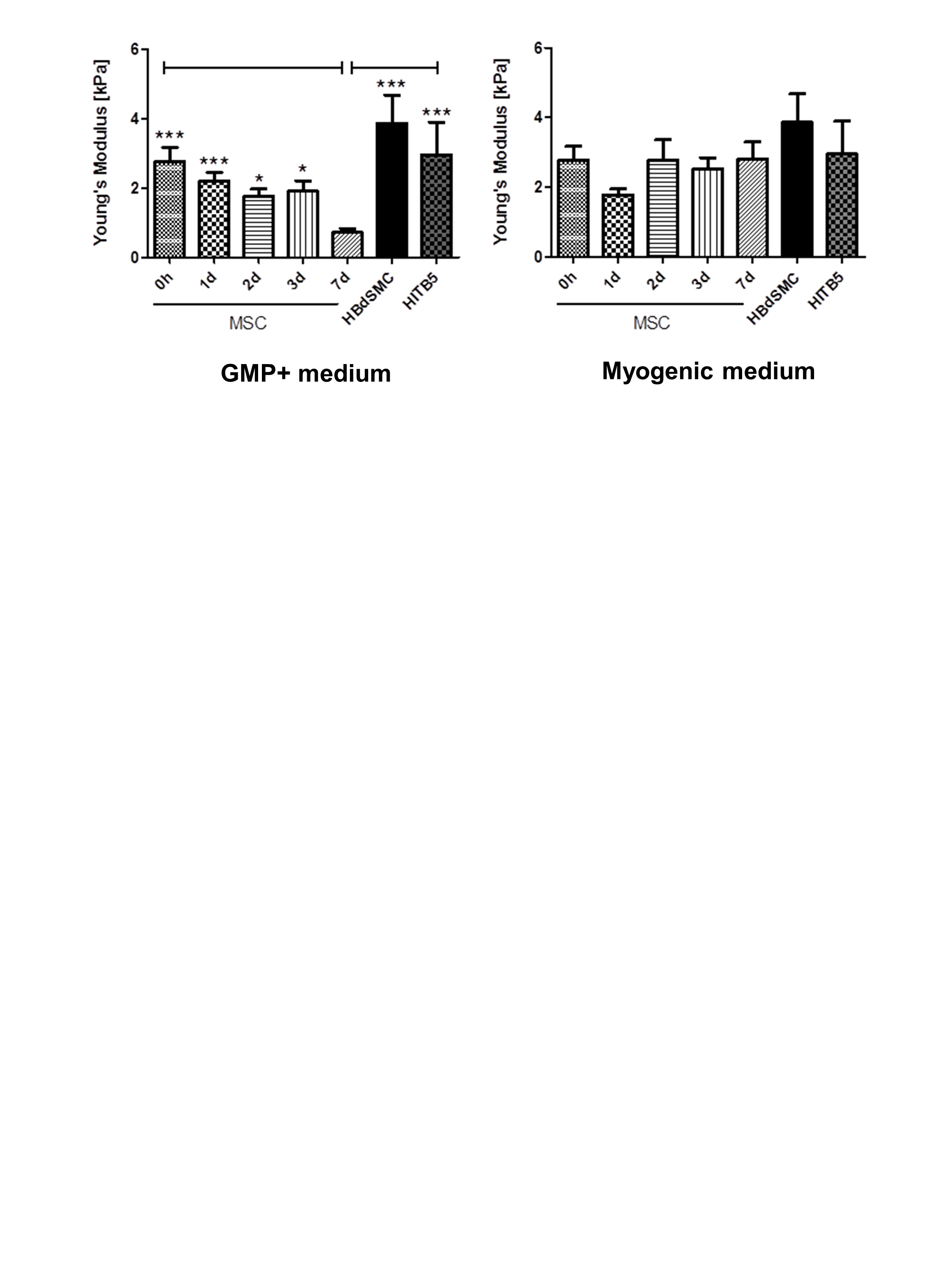

Supplement: S5 Fig — Young’s modulus as a measure of the stiffness of the cells was determined for MSCs cultured in expansion media (GMP+). During the seven days of culture these cells became softer, in contrast to MSCs cultured in myogenic differentiation medium. For comparison the elasticities of HBdSMC and HITB5 were determined. (n = 3 donors; error bars indicate standard error of the mean; one-way ANOVA analysis; *p<0.05; ***p<0.001). (TIF) [file pone.0137419.s005.tif]

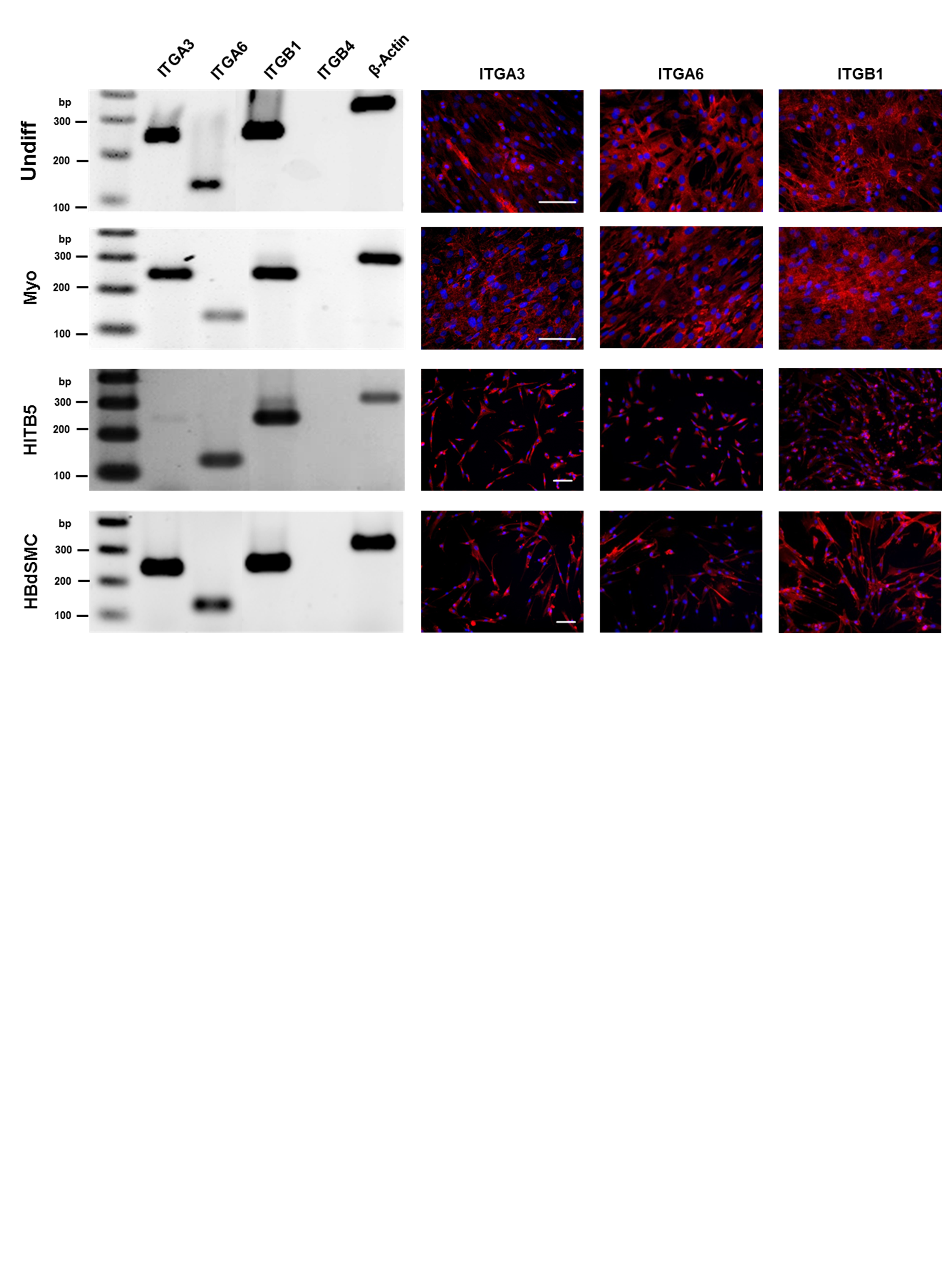

Supplement: S6 Fig — RT-PCR analyses and immunofluorescence staining of undifferentiated MSCs (Undiff), myogenically differentiated MSCs (Myo), HITB5 and HBdSMC indicated the expression of several laminin-binding integrin receptors. The integrin-α3 chain (ITGA3), the integrin-α6 chain (ITGA6) and the integrin-β1 chain (ITGB1) were strongly expressed by all analyzed cell types. The integrin-β4 chain (ITGB4) was not expressed by these cells. Cell nuclei were counterstained in blue with DAPI (bars: 100 μm). (TIF) [file pone.0137419.s006.tif]

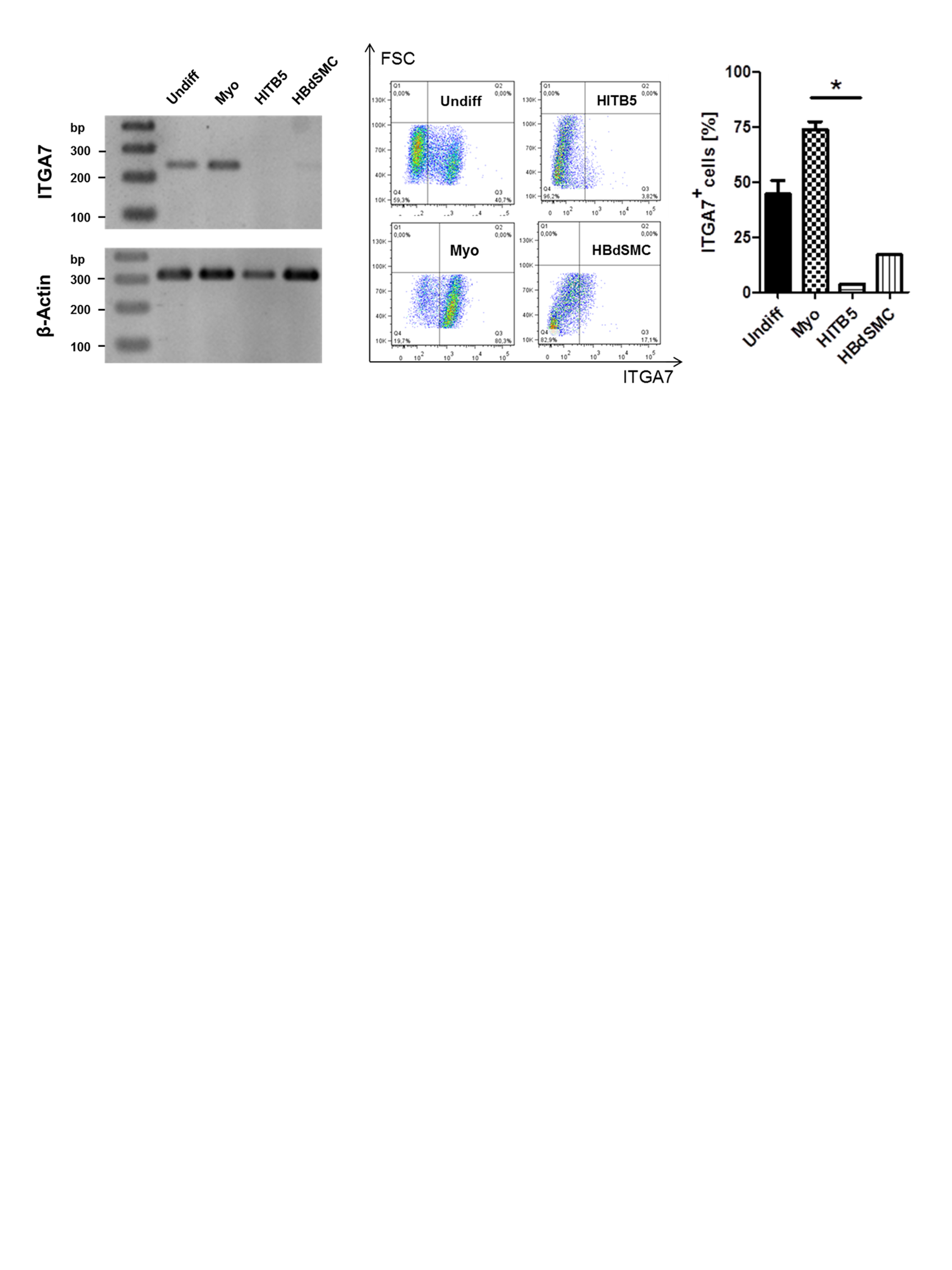

Supplement: S7 Fig — RT-PCR and flow cytometry analysis showed the expression of the integrin-α7 chain on undifferentiated MSCs (Undiff) and myogenically differentiated MSCs (Myo), but not or almost not on HITB5 and HBdSMC. The highest expression was observed for myogenically differentiated MSCs. Undifferentiated MSCs expressed ITGA7 at an intermediate level (n = 3 donors; error bars indicate standard error of the mean; t-test analysis; *p<0.05). (TIF) [file pone.0137419.s007.tif]
